# Using Innovation-Corps (I-Corps™) Methods to Adapt a Mobile Health (mHealth) Obesity Treatment for Community Mental Health Settings

**DOI:** 10.3389/fdgth.2022.835002

**Published:** 2022-05-27

**Authors:** Rita Haddad, Carolina Badke D'Andrea, Amanda Ricchio, Bradley Evanoff, Elaine H. Morrato, Joseph Parks, John W. Newcomer, Ginger E. Nicol

**Affiliations:** ^1^Healthy Mind Lab, Department of Psychiatry, Washington University School of Medicine, St. Louis, MO, United States; ^2^Center for Healthy Work, Department of Internal Medicine, Washington University School of Medicine, St. Louis, MO, United States; ^3^Parkinson School of Health Sciences and Public Health, Loyola University of Chicago, Chicago, IL, United States; ^4^Colorado School of Public Health, University of Colorado Anschutz Medical Campus, Aurora, CO, United States; ^5^National Council for Mental Wellbeing, Washington, DC, United States; ^6^Thriving Mind South Florida, Miami, FL, United States

**Keywords:** mentally ill persons, health services, implementation science, innovation-corps, clinical and translational science, obesity

## Abstract

**Background:**

We employed Innovation Corps (I-Corps™) methods to adaptation of a mobile health (mHealth) short-message-system (SMS) -based interactive obesity treatment approach (iOTA) for adults with severe mentall illness receiving care in community settings.

**Methods:**

We hypothesized “jobs to be done” in three broad stakeholder groups: “decision makers” (DM = state and community clinic administrators), “clinician consumers” (CC = case managers, peer supports, nurses, prescribers) and “service consumers” (SC = patients, peers and family members). Semistructured interviews (*N* = 29) were recorded and transcribed ver batim and coded based on pragmatic-variant grounded theory methods.

**Results:**

Four themes emerged across groups: education, inertia, resources and ownership. Sub-themes in education and ownership differed between DM and CC groups on implementation ownership, intersecting with professional development, suggesting the importance of training and supervision in scalability. Sub-themes in resources and intertia differed between CC and SC groups, suggesting illness severity and access to healthy food as major barriers to engagement, whereas the SC group identified the need for enhanced emotional support, in addition to pragmatic skills like menu planning and cooking, to promote health behavior change. Although SMS was percieved as a viable education and support tool, CC and DM groups had limited familiarity with use in clinical care delivery.

**Conclusions:**

Based on customer discovery, the characteristics of a minimum viable iOTA for implementation, scalability and sustainability include population- and context-specific adaptations to treatment content, interventionist training and delivery mechanism. Successful implementation of an SMS-based intervention will likely require micro-adaptations to fit specific clinical settings.

## Introduction

Obesity is two to three times more prevalent in people with severe mental illness (SMI), contributing to higher rates of obesity-related conditions like type 2 diabetes and cardiovascular disease (CVD) compared to the general population (1). This burden of cardiometabolic risk contributes to a 10–15 year mortality gap between those with SMI and the general population (2). Obesity treatments that have been tested in real-world treatment environments, like community mental health clinics (CMHCs) and Clubhouse settings where SMI is routinely treated, consist of group or individual counseling sessions delivered either by clinicians or peer health coaches (3, 4). While most interventions are associated with some health behavior change or improved self-efficacy, most fail to separate from controls on a primary outcome of weight loss (5–8). Further, reduction or reversal of weight loss achieved during active treatment is observed as soon as 2 months post-intervention (7), suggesting the need for maintenance treatment in real-world clinical settings (9).

Community-based mental health care is associated with better medical monitoring and treatment engagement in people with SMI (9–11). Many obesity interventions are designed to leverage exisiting resources by relying on clinical staff trained in health coaching (12–14). However, obesity and cardiovascular disease risk are part of a long list of clinical issues that must be addressed by clinical staff, creating significant barriers to successful implementation (15, 16). We aimed to adapt an existing interactive obesity treatment approach (iOTA) (17–19) employing short message system (SMS) texts, a highly utilized technology among low-income and mentally ill populations (20). Semi-automated support texts and weekly prompts to self-monitor weight and goal progress provide opportunities for increased engagement by consumers of clinical services, extending the reach of in-person health coaching (21, 22).

**Figure 1:**
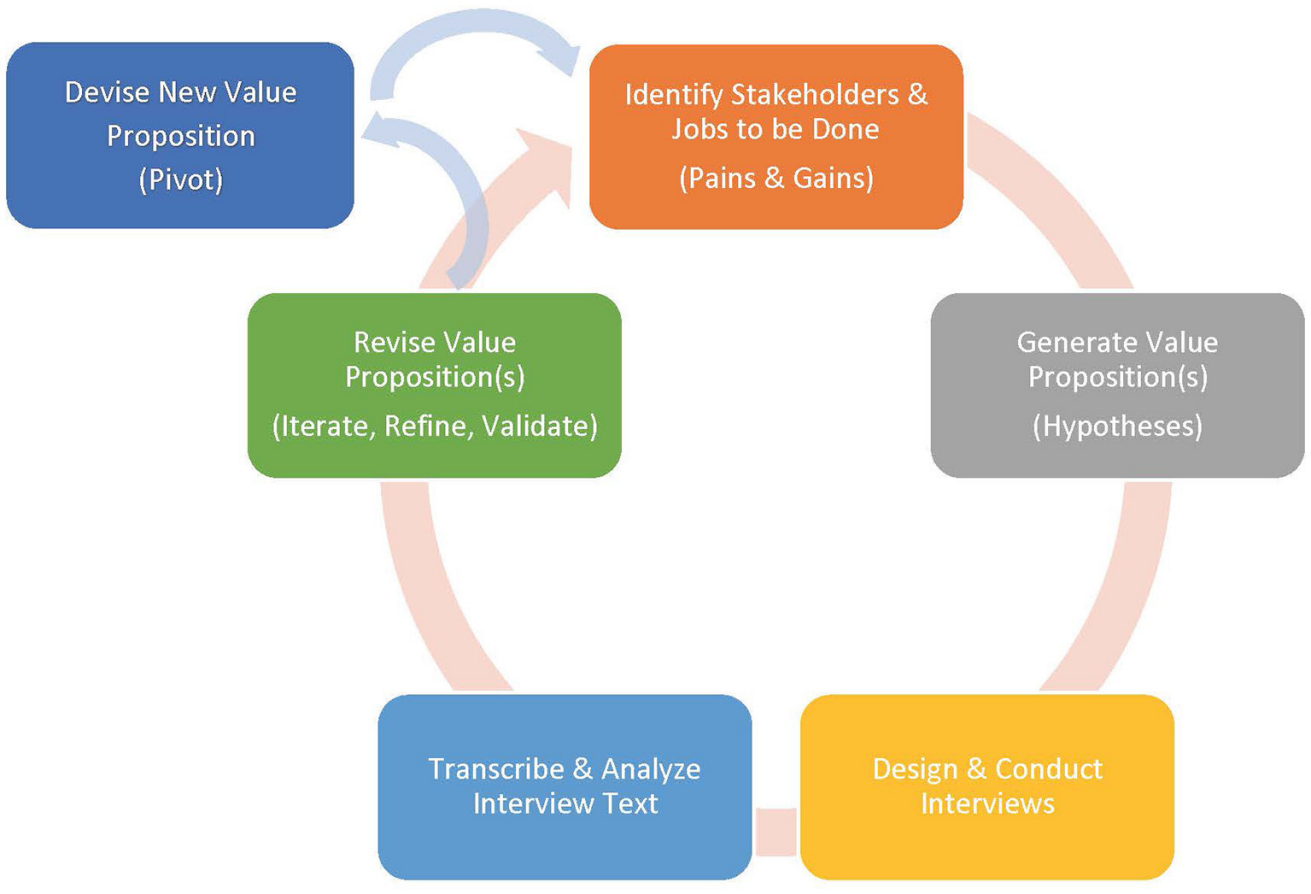
Innovation corps process.

Each CMHC and Clubhouse ecosystem is unique, meaning health promotion programs must be tailored both to the clinical population and treatment setting in order to be successful (23). Thus, multi-level stakeholder engagement is critical for adapting or developing programs before they are implemented (24, 25). Innovation Corps (I-Corps™) methodology, co-created by the National Science Foundation (NSF) (26) and adopted by the National Institutes of Health (NIH), (27) uses the design-based Lean Launchpad approach, popularized for tech startups for the academic research audience to facilitate customer-centered design and promote more successful commercialization of technology and engineering innovation into real-world settings and markets (28, 29). The emphasis of the I-Corps method is on immediate and iterative collection of stakeholder feedback via “customer discovery” interviews to identify and validate a “value proposition” for key consumers (Figure 1). From this, major “gains,” “pains” and “jobs to be done” are identified, and used to revise assumptions and hypotheses, testing redesigned offerings and making further small adjustments (iterations) or more substantive ones (pivots) to improve outcomes.

In the present study, we employed I-Corps methods to identify challenges or “pains” associated with wellness program implementation in CMHC and Clubhouse settings. In general, the project team hypothesized that some areas of concern would be thematically unified across stakeholder groups. We also expected that certain barriers faced by each group would be unique, with overlap between stakeholders with similar roles. In order to allow for a broader interview framework, we formed our hypotheses based on stakeholder groups condensed into three broad categories: decision makers (DM; state and local clinical administration), clinician consumers (CC; nurses, prescribers, community support workers, case managers, psychosocial rehabilitation counselors, peer supports) and service consumers (SC), related to successful implementation of hybrid health coaching interventions targeting weight management via lifestyle change. We hypothesized that barriers for the DM groups would include limited resources to support implementation, while CC groups would experience barriers relative to burnout and mismatch between administrative expectations and clinician reality. Lastly, we expected barriers at the SC level to include illness-related difficulties managing the increased cognitive load associated with learning and practicing new health behaviors, leading to reduced motivation and engagement.

## Methods

I-Corps methodology consists of four steps: (1) identify the relevant target consumers and decision-makers as key stakeholders, (2) identify potential unmet needs or issues in the existing services or products relative to available alternatives, including the *status quo*, (3) create testable and pliable “value proposition” hypotheses for the proposed products or services solutions based on these unmet needs, and (4) conduct customer-stakeholder interviews to identify and validate which problems resonate as the most critical or most important to solve (Figure 1).

Five settings in Missouri and Florida were identified for discovery—two CMHCs treating children, adolescents and adults, one Clubhouse, and two ambulatory academic teaching clinics, one treating children and adolescents, and one treating adults. Purposive sampling methods were used to identify relevant stakeholders with diverse perspectives on technology, community mental health care and healthy lifestyle interventions (30). Specifically, the study team built upon existing relationships with clinical leadership and knowledge of the clinical settings to recruit for interview and focus group participants. Clinical leadership were asked to provide recommendations for stakeholders, as well as introductions to leaders in each stakeholder group.

Based on the clinical structure and hierarchy in most community clinical settings, and in consultation with clinical leadership in each setting, we identified 6 broad stakeholder groups across the three categories: “Service Consumers,” included ambulatory outpatients, CMHC and Clubhouse members, (*n* = 4); “Clinician Consumers,” included nurses (*n* = 4), psychosocial rehabilitation (PSR) counselors, community support workers, case mangers, social workers (*n* = 5), and prescribing clinicians (attending, resident and fellow physicians, advance practice nurses) (*n* = 8); “Decision Makers,” included local clinic and clubhouse administrators (*n* = 5), and state administrators (*n* = 3).

We hypothesized that each stakeholder group would have unique jobs and associated barriers when engaging clinical service consumers in health behavior change related to weight management, but that the use of simple technology to provide reminders and reinforce goal-oriented health behavior change would be viewed favorably as a tool for providing enhanced support to clinical service consumers compared to existing practices thereby extending the clinician's impact without increasing work burden or cost. From this hypothesis, we generated an initial value proposition statement: “*an iOTA, in combination with existing health coaching approaches, can help clinicians more effectively engage consumers of clinical services in health behavior change compared with the leading alternative of counseling on energy-balance (e.g., calories consumed versus calories burned) delivered by staff with limited training or programmatic structure.”*

We then created a brief semi-structured interview (Table 1) to identify stakeholder attitudes and beliefs about weight managemet (is it a priority “job to be done”?), experience with selecting and executing wellness interventions, including benefits or benchmarks of success (“gains”) and barriers or challenges to successful implementation (“pains”). Follow-up questions were based on the research team's prior knowledge of the CMHC settings, as well as review of the existing implementation research in this area (15).

**Table 1:** Semi structured interview questions and probes.

**Domain**	**Question**	**Examples, clarification,** **follow-up probes**
Program selection and implementation	How do you decide what evidence-based interventions to implement in your setting?	•What holds you back from implementation? •What would be needed to change this?
Insitutional memory of change	Can you remember a time when [you/your organization] implemented a health behavior change program?	• What was successful/good about it? • What challenges did you have?
Wellness values	Is physical health and lifestyle change important to [you/your organization]?	• If yes, how do you discuss it (with peers, providers, or supervisors)? If no, why? • What do those around you say/do?
Use of technology	What do you think about the use of technology (mHealth applications, text messaging, telehealth) for delivering health care?	• What are the barriers to using technology for supplementing clinical care in your setting? • What are the advantages or barriers to using technology to support lifestyle change programs?
Change culture/values	What are the biggest barriers to implementing a health behavior change program in your care setting?	• What challenges have you faced making health behavior changes the past?
Perceptions of research and academia	Would you/your organization be willing to participate in a randomized study of such an intervention?	• Would having a “health coach” in addition to a case manager or nurse be helpful or unhelpful? • How would you feel about a research study that randomized to a control condition, like health education?

### Data Analysis

Interviews were continued until thematic saturation was achieved and were recorded and transcribed verbatim. Transcripts were analyzed using an inductive coding approach based on pragmatic-variant grounded theory (31). The first and second authors (RH and CD) independently identified emergent codes related to barriers to and facilitators of successful health behavior change programming across stakeholder groups. This initial set of coding was reviewed by the senior authors (GN and JN) to identify and remove unclear and redundant codes, or discrepancies, and to evaluate for any bias. Reliability in the initial coding phase was achieved through consensus discussions and related code modifications until consensus was reached. Revisions to hypothesized “jobs to be done”, “pains” and “gains” for each stakeholder group were based on prevalent themes and reviewed for consensus among members of the research team before being included in the final data set. The final set of themes and subthemes were then used to revise the value proposition. Nvivo-12 (32) software was used to track coding notes, changes, categories, and frequencies of codes and quotations.

## Results

Twenty-nine stakeholder participants were identified within the selected clinical settings and consented to participate in the study. The breakdown of characteristics by stakeholder group can be found in Table 2. *Four* broad themes emerged across stakeholders. These included Education, with sub-themes of existing knowledge and awareness, professional training, and clinicians as educators; Inertia, with sub-themes of symptom severity, readiness for change, motivation, avoidance and overwhelm; Resources, with sub-themes of scarcity in financial resources, social support, referral resources, access to psychological support and consideration of mobile technology as a resource; and Ownership, with sub-themes of accountability, preference for individual vs. shared process ownership, and staff empowerment. Unique jobs to be done, pains and gains were identified in each stakeholder group. Representative quotes and number of mentions within each sub-theme, by stakeholder group are listed in Table 3 and summarized in detail below based on stage of the I-Corps customer discovery process.

**Table 2:** Participant characteristics.

**Role**	**Care setting**	**Gender**	**Race/** **ethnicity**
**State administrators (DM) (*****n** **=*** **3)**			
Manager of integrated care	CMHC	F	White
Director of clinical operations	CMHC	F	White
Integration health manager	CMHC	F	White
**Local clinic and clubhouse administrators (DM) (*****n** **=*** **5)**			
Clinic manager	Child psychiatry teaching clinic	F	White
Director of nursing	CMHC	F	Black
Clinical director	CMHC	F	Black
Team supervisor	CMHC	F	White
Healthcare home director	CMHC	F	White
**Prescribing clinicians (physicians, advance practice nurses) (DM and CC) (*****n** **=*** **8)**			
Child psychiatry fellow (Post graduate year-5) (CC)	CMHC and child psychiatry teaching clinic	M	Asian
Child psychiatry fellow (Post graduate year-4) (CC)	CMHC and child psychiatry teaching clinic	M	White
Psychiatry resident (Post graduate year-3) (CC)	CMHC, child and adult psychiatry teaching clinics	M	White
Training director (DM)	Child psychiatry teaching clinic	F	White
Clinic director (DM)	Child psychiatry teaching clinic	M	White
Clinic director (DM)	Adult psychiatry teaching clinic	M	Black
Medical director (DM)	CMHC	M	White
APRN (CC)	CMHC (FL)	F	White/Hisp
**Nursing staff (CC) (*****n** **=*** **4)**			
RN	Adult psychiatry teaching clinic	F	Black
RN	CMHC	F	White
RN	CMHC	F	Black
RN	CMHC	F	White
**Psychosocial rehabilitation counselors (Community support workers, case managers, social workers) (CC) (*****n** **=*** **5)**			
Social worker	Adult psychiatry teaching clinic	F	White
Case manager	CMHC	F	White
Case manager	CMHC	F	White
Social worker	CMHC (FL)	F	White/Hisp
Case manger	CMHC (FL)	F	White/Hisp
**Service consumers (CMHC and clubhouse members) (SC) (*****n** **=*** **4)**			
Young adult (<30 years)	Adult psychiatry teaching clinic	M	White
Adult (30-65 years)	Adult psychiatry teaching clinic	F	White
Adult (30-65 years)	CMHC	M	Black
Young Adult (<30 years)	CMHC	F	Black

*CC, Clinician Consumers; SC, Service Consumers; DM, Decision Makers*.

**Table 3:** Subthemes and representative quotes by stakeholder group.

	**State administration**	**Local clinic administration**	**Prescribing clinicians**	**Nurses**	**Psychosocial rehabilitation (PSR) counselors**	**Consumers of clinical services**
**Education**	***Staff training: (10 mentions)*** “We're really trying to help behavior health professionals understand their place at the table when it comes to addressing physical health with their patients.” “We bring staff in annually to provide training…it's a train the trainer [model], then those folks go back and train the staff and the agency on wellness coaching.”	***Prescriber awareness and training: (5 mentions)*** “I think probably the thing holding [implementation] back is frankly ignorance about what exists.” ***Staff education: (18 mentions)*** ***“***if we train staff to really know how to manage and work with a particular group, they tend to do better with them because they don't have to be a jack of all trades.”	***Prescriber awareness and training: (45 mentions)*** ***“***the only training you get about weight management is one lecture during residency” “That's how I was trained. The goal was to kind of enhance your motivational interviewing skills to be able to use those to maybe talk more to the kids about drugs and stuff like that and how to quit.”	***Nurses as educators: (26 mentions)*** ***“***we have our own way of educating… We're constantly talking about you know the importantance of being physically active, making healthy choices.” “We love when the caseworkers are there because we're educating them to help educate the patients.” “When new case managers come in, they're educated from the start that we take care of the whole person.”	***PSR Counselor knowledge/training: (19 mentions)*** “Nowhere in my training were they like, ‘and in addition to assessing for all of their resources and their psychosocial stressors and all of that, you also need to talk to them about their lifestyle habits'.” ***Patient awareness and engagement: (21 mentions)*** “A lot of workers probably would be fine with implementing those types of programs if they could get a response from the patient in being able to attend, participate, and engage.”	**Consumer knowledge (13 mentions):** “It's a lack of education on what to eat…I don't know when to say when.” “I'm trying to figure out how to get a happy medium where I'm not eating the wrong stuff and trying to find a healthy lifestyle.” “I've dealt with this for most of my life. How I can stop from eating too much and start living more healthy?”
**Inertia**	***Stability of patient mental health: (3 mentions)*** “We have to be very cognizant that [patients] sometimes don't know where they're going to sleep tonight.” ***Staff workload: (2 mentions)*** “our (staff) have been overwhelmed with visits, so the climate is not good for us to try to come in and have them do more.”	***Overwhelming clinical demands: (9 mentions)*** “When you're dealing with symptoms, housing issues, financial issues, all these competing things, it's hard to worry about your cholesterol level.” ***Patient readiness for change: (18 mentions)*** “People's readiness for change can move. You could start, something may occur in your life…that may throw you off track for a while.”	***Prescriber workload: (19 mentions)*** ***“***There's so many things competing for your time and energy over a 30 minute appointment.” ***Patient motivation: (59 mentions)*** “Are they going to follow through, is the patient a good fit for it, does it require a lot of time? Do I think they're going to do it?” ***Patient shame and avoidance: (15 mentions)*** “(Patients) don't bring it up and whenever you even insinuate stuff about [weight], they shut down.”	***Patient motivation: (20 mentions)*** “A lot of our patients, because of their illnesses, have very poor motivation” “This is just something else, they think ‘I'll read this later' and you know life goes on and they don't look at it.” ***Competing clinical priorities: (24 mentions)*** “Sometimes the mental health stuff takes precedence and they forget about diabetes or seeing a doctor for their blood pressure.”	***Patient priority and motivation: (26 mentions)*** “(Patients) aren't as willing to put forth effort. They're just kind of like ‘this is a lost cause.' They know they can't really do it on their own.” ***Staff priority and workload: (48 mentions)*** ***“***Nobody has time to do it well. Case loads have gotten pretty high.” “(Caseworkers) refer patients to services knowing that it would be like a relief for them to have their patient involved.”	***Patient avoidance: (9 mentions)*** “[Talking about my weight]…makes me uncomfortable” “When I get off of work I just want to eat and crash.” ***Patient motivation: (24 mentions)*** “By the time I get to the gym, I don't feel like being there.” “Another thing that got discouraging was that I'd work out and not lose any weight. I was like ‘What is the point again?'”
**Resources**	***Organizational financial resources: (8 mentions)*** ***“***One of the problems with us providing training is that our funding from year to year is never the same” “[A selected wellness program] has got to be something that is	***Organizational financial resources: (25 mentions)*** “A big barrier to implementing anything is finding a funding source that will pay for it. Even if we got a dietician…you're talking about a massive amount of money.”	***Patient financial resources: (15 mentions)*** “It's not just cost of healthy food, but accessibility to it.” “Logistics is a big issue…It's not realistic for them if they live two hours away to come back a couple of weeks later.”	***PSR Counselor overwork: (18 mentions)*** “Their caseloads are huge…we're often short staffed with caseworkers.” “The case workers are overloaded. If we weren't as short staffed, then the	***Patient financial resources: (50 mentions)*** “Let's say they can take the bus. Are they able to hold all of their groceries or are they going to have to decide they're only going to get five bags of groceries verses 10?”	***Patient financial resources: (14 mentions)*** “Financial is a big part because the cheapest foods are the crappiest foods” “Low income families, you know, it's like they can't afford to buy the good stuff.”
	not going to cost a ton of money and that there's not licensing fees… sustainability is really what guides us [in chosing programming].”	***Patient financial resources: (3 mentions)*** “The fact is that sometimes you can't get a hold of our population. Some of them are homeless and some of them don't even have telephones.”	***Patient family support: (7 mentions)*** “Parents will probably be a big factor. Usually their own time is pretty limited.” ***Prescriber openness to mHealth as a resource: (15 mentions)*** “I still think that they're more likely to follow up, you know because they're getting texts and we're not asking them to come in every week.”	caseworkers probably would be able to provide more quality service.” ***Need for outside health promotion resources: (10 mentions)*** “I can give them the information and highlight things like phone numbers, important people to call with questions… “Having [a referral resource] who is really knowledgeable beyond basic caseworker or nurse knowledge of things would be helpful.”	***Organizational financial barriers: (11 mentions)*** “There would have to be like a financial incentive. They wouldn't let somebody start (a new program) if there wasn't going to be a way to bill for it.” ***PSR Counselor openness to mHealth as a resource: (12 mentions)*** “If they could see or interact with [a program] on their smartphone, that would be easier for them to access than actually having to leave their house and go to a group.”	“I tried going to the gym but that didn't work because it was further away from my home” ***Patient access to emotion regulation/support: (21 mentions)*** “I need a lot of reassurance. If maybe someone called once a week and said how are you doing, that would help.” ***Patient openness to mHealth as a resource: (7 mentions)*** “[Text messaging] would be appealing for me because you almost feel like you're being held accountable.”
**Ownership**	***PSR Counselor accountability: (17 mentions)*** “We repeat to them over and over again that what it boils down to is behavior changes are needed and to manage the condition, no matter what the condition is…as behavior health specialists, that is exactly what they went to school for.” ***Organizational accountability: (16 mentions)*** “Some agencies have completely gotten on board with it…others do a very brief training for their staff and it just doesn't really take off there. So it's kind of a mixed bag.” ***State administration accountability: (21 mentions)*** ***“***We recognize the need to have a fidelity component built in…we've offered support with one on one coaching with a trainer and ongoing webinars.”	***Local Administration ownership: (23 mentions)*** “Right now, my primary thing is bringing groups together and looking at them and what they can do so they can be more efficient for patients.” ***Case manager ownership: (32 mentions)*** ***“***You have to get [staff] that's dedicated enough to do it and wants to do it…case managers are going to feel like it's just another thing because they're already doing a lot.” ***Prescriber ownership: (18 mentions)*** “Medical providers getting on board and obtaining the right education and knowing the right processes and staying current are the biggest things…. Continued education is all on them.”	***Prescriber ownership: (35 mentions)*** “I don't let go of wanting to address the problem just because people are not ready.” ”I definitely monitor your weight but it's definitely more of a red flag when I think it's a medication side effect because that means I caused it.” ***Prescriber preference for shared ownership: (15 mentions)*** “What you have to have happen for a successful program would be to have a person who has more time to devote to creating and implementing it.” “In years past, I had a social work student who was working with me and I had her work on nutrition educational modules where she could spend half an hour talking to some of my patients…They really loved it because they were getting one on one attention and they felt pampered.”	***Nurse preference for shared ownership: (38 mentions)*** “We really work well together as a team.” “We [nurses] see her weight is going up, I know about it, now it's on me. I need to make sure that I'm encouraging her to talk to the doctor.”	***PSR Counselor preference for shared ownership: (48 mentions)*** “I pull my nurse in and let them explain what's happening in the body and they are really good at breaking that down and making it easy to understand…because it's not my strength.” “Sometimes people want to hear it from a nurse and not a social worker.” ***PSR Counselor empowerment: (11 mentions)*** “As a worker, I wouldn't feel like it would really be my place to recommend something like that.” “I'm not a nurse. I'm not a doctor. So I really use the resources I have internally and learn from them so that I do have more knowledge.”	**Patient accountability: (29 mentions)** “I always go to the store and get the wrong thing that's not good for me. I just make poor choices.” “Maybe if I had an accountability partner or something like that. Somebody that would keep me accountable.”

### Consumer Jobs to Be Done

#### State Administrators

State administrators considered physical and mental health to be of equal importance, and viewed their primary jobs related to physical health and weight management of clinical consumers as (1) selecting physical health and wellness programs for statewide implementation based on needs identified by local clinical administration, (2) providing staff training for dissemination, and (3) ensuring sustainability of implementation with ongoing fidelity monitoring.

#### Local Clinic Administrators

Local clinic administrators acknowledged the importance of addressing SC physical health in balance with acute SC care needs and staff workload, and perceived their primary responsibilities as promoting clinician accountability for professional development, fostering a workplace culture of team-based clinical care, and protecting clinician time for SC care.

#### Prescribing Clinicians

Prescribing clinicians felt a responsibility to help treatment teams prioritize the often competing physical and mental health demands in order to determine which SCs would benefit from or be likely to engage in an intervention, support the clinician providing health coaching and reduce health risks by optimizing medical monitoring and management.

#### Nurses

Nurses embraced responsibility for supporting physical health of SCs, and considered themselves as process owners who prioritize organizational values regarding physical health and wellness. Among their primary job functions, nurses supported medical recommendations via education and coaching to promote health behavior change.

#### Psychosocial Rehabilitation Counselors

PSR counselors were the clinicians most likely to be assigned health coacing roles in each setting. They viewed themselves as process owners for implementation of the treatment plan, focused on optimizing psychosocial functioning and quality of life, and addressing acute psychosocial concerns (e.g., housing, legal concerns). However, this group did not believe they were best suited to own the process of addressing physical health, including the implementation of weight management programming, which was viewed as separate from the overall treatment plan.

#### Service Consumers

SCs indicated that their greatest needs were in accountability, particularly in staying engaged with self-monitoring. Thus, as physicians noted engagement as a reason for their inertia in delivering behavioral lifestyle counseling, SCs also noted this as a problem—one they wanted help with.

### Consumer Barriers or “Pains”

#### State Administrators

State administrators noted that many available health and wellness programs required financial commitments for ongoing training or licensing (education, resources). Inconsistent program implementation at the local level was attributed to turnover or attrition of staff trained as trainers, and to limited time to participate in ongoing training to ensure fidelity of intervention delivery (resources, education). Digital and mobile health (mHealth) interventions were perceived as potential barriers to adoption of new interventions due to variability in use of technology from setting to setting, with equally varied attitudes toward technology within each unique clinical setting.

#### Local Clinic Administrators

Local clinic administrators noted that although training offered by the state was free, clinics were still responsible for covering the time away from SC care to accommodate training and trainer activities. In absence of resources to make up for loss of revenue-generating activities, directors were tasked with identifying existing resources to support program implementation and sustainability.

#### Prescribing Clinicians

Prescribing clinicians in teaching roles expressed confidence in the use of motivational interviewing for promoting healthier behaviors, while physician trainees were less confident, indicating limited didactic or experiential learning on the subject. Both groups acknowledged implementation challenges related to process ownership and SC engagement.

#### Nurses

Nurses were most likely to either create resource lists or design their own coaching approach with SC in settings where internally available programs were not available. Limited time and competing clinical demands posed the biggest challenges to implementation.

#### Psychosocial Rehabilitation Counselors

PSR counselors, though trained as health coaches in many settings, felt less secure in their medical knowledge, and were reluctant to accept ownership of this role. Attrition of staff who led original trainings lead to degradation of treatment delivery over time.

#### Service Consumers

SCs noted that lack of emotion regulation skills and social supports reduced their resolve to make healthy choices. They indicated a need for problem-solving help with pragmatic issues such as where to buy fresh produce with limited money, how to cook healthier foods and how to make healthy selections when not eating at home (e.g., at the Clubhouse cafeteria).

### Consumer Benefits or “Gains”

#### State Administration

State administration considered successful training and program implementation in terms of SC-level health outcomes, as well as in workforce development. Employing a “train the trainer” model was seen as a way to reduce training costs while empowering local organizations and staff, increasing uptake and organizational culture change. The use of semi-automated SMS texting was seen as a possible augmentation to existing care to extend clinician reach and increase SC engagement.

#### Local Clinic Administration

Local clinic administration viewed collaboration with academic researchers as a career development opportunity for staff and an opportunity to enhance clinical care. Administrators considered mobile technology as useful in concept, but saw potential barriers to implementation in terms of integration with the current work flow for clinicians. Subthemes under ownership also involved protection—of SCs (safety) and of staff (from additional work burden).

#### Prescribing Clinicians

Prescribing clinicians identified SC engagement as a major contributor to successful treatment outcomes, impacted by intrinsic motivation or incentive, shame/avoidance, and previous negative experiences—both from the perspective of the physician (limited perceived benefit for additional work burden) and the SC/family (limited perceived benefit for additional burden of time and mental energy). In general, physicians viewed mobile technology as a potential solution to the additional work burden and to SC engagement in wellness programming.

#### Nurses

Nurses noted they could fill in gaps when case managers were dealing with competing psychosocial clinical priorities that took precedence over health prevention measures. In settings where wellness programming was available, nurses consistently refered SCs to those programs and seemed to have the most knowledge about them. Nurses cautiously perceived mobile technology as a way to increase engagement in wellness programming, but did not feel this could take the place of education or clinical care.

#### Psychosocial Rehabilitation Counselors

PSR counselors preferred a team approach to delivering health interventions with their SCs, and perceived the use of technology to extend their reach to SCs as potentially helpful as long as this respected therapeutic boundaries or safety of personal data.

#### Service Consumers

SCs indicated an openness to the use of mHealth technology to increase their sense of external support and accountability, indicating that they would be most comfortable with a case manager in the role as health coach.

### Evolution of the Value Proposition

Similarities in priorities across groups were observed (Table 3). Administartors wanted a sustainable, effective and low-cost intervention. Nurses asked for an intervention that takes into consideration the dynamic needs of the SC population and emphasizes team-based care. PSR counselors supported an intervention that would help them and their SCs without adding a time burden, and were supportive of mHealth for this purpose. Physicians wanted to be able to refer to a behavioral weight loss program and actively track their pateint's results. SCs wanted a simple intervention that addresses energy balance while providing emotional support. With this insight, our overall value proposition became “*Enhancement of existing wellness programming with low-cost text messaging support can promote health behavior change in SCs with SMI better than the current leading alternative of counseling on energy balance delivered by staff with limited training.”*

## Discussion

Using I-Corps, a novel stakeholder-centered method for designing health intervention for dissemination and sustainabilitys, our results suggest adjustments to the current health coaching delivery model are needed for successful implementation in community setting. In particular, case manager time constraints and challenges to successful SC engagement were cited as significant barriers to implementation, with most respondents expressing a belief that technology could be used to simplify treatment delivery and improve engagement. Additional barriers to uptake included role confusion (e.g., which clinician holds ownership of the process), fragmented communication between providers, and low motivation for change among SCs. This study provides critically-needed information for the successful adaptation of obesity treatment for the SMI population, addressing contextual factors relevant to the CMHC and Clubhouse settings.

The four major “barrier” themes (resources, education, inertia and ownership), while similar across groups, were interpreted differently based on stakeholder “jobs to be done.” A final fifth barrier was identified, involving discrepancy in who should function as the health coach. Decision makers identified PSR counselors and/or case managers as being the best positioned to function as health coaches, given their proximity and rapport with SCs. Nonetheless, this group was reluctant to accept ownership of this role. These results extend previous work suggesting that barriers to successful treatment engagement are multifactorial, involving specific challenges at the SC, provider and organizational levels (33). However, few studies have explicitly evaluated barriers specific to treatment setting, including the perspective of SCs, potential interventionists, treatment team leaders and decision-makers (34).

The use of technology to extend the reach of health coaches was seen as promising by both clinicians and SCs. Our results suggest adaptations to traditional health coaching delivery models, where existing clinical staff are trained and responsible for implementation, may benefit from the introduction of technology. These results are consistent with previous reports in SMI populations suggesting openness to the use of technology for delivering health behavior change interventions, which have been shown to boost treatment engagement in non-mentally ill obese adults (1, 35, 36).

The present study is subject to strengths and limitations. First, the I-Corps approach is a proven methodology consistent with best practices for designing for dissemination and diffusion of health innovations (37, 38). Though we perceive interviewing stakeholders at different settings in two states as a major strength, this approach does not fully address limitations to the generalizability of our results. Grouping all mental illnesses under “SMI”, and interviewing only four SCs also limits generalizability for specific diagnoses. Finally, using a qualitative process that relies heavily on self report subjects our data to reporting bias. Despite these limitations, this study also has several innovative aspects that strengthen the qualitative research methods. First, we employed an evidence-based, holistic mixed-methods approach (39, 40), including clinical use validation, to anticipate context-specific treatment adaptations ahead of implementation. This is a critical step in translating evidence-based interventions into real-world settings. We also employed gold-standard sampling methods and coding techniques, as well as strengthened rigor and reproducibility by engaging in consensus coding exercises (41, 42). In summary, this study demonstrates the application of novel qualitative methodology as first steps toward adapting and implementing weight management interventions in community mental health treatment settings.

## Data Availability Statement

The raw data supporting the conclusions of this article will be made available by the authors, without undue reservation.

## Ethics Statement

The studies involving human participants were reviewed and approved by Washington University Institutional Review Board. The patients/participants provided their written informed consent to participate in this study.

## Author Contributions

All authors listed have made a substantial, direct, and intellectual contribution to the work and approved it for publication.

## Funding

Research reported in this publication was supported by the National Center for Advancing Translational Sciences of the National Institutes of Health under Award Numbers UL1TR002535, UL1TR003096, UL1TR001417, and the National Institute of Mental Health Grant Numbers MH112473 and MH118395. This work was also made possible by Public Health Cubed seed funding via the Washington University School of Medicine Institute for Public Health.

## Author Disclaimer

The content is solely the responsibility of the authors and does not necessarily represent the official views of the National Institutes of Health.

## Conflict of Interest

BE has received funding from the National Institutes of Health (NIH) and the Centers for Disease Control and Prevention (CDC). EM has received grant support from the National Institutes of Health (NIH) and the CDC and has consulted for the U.S. Food and Drug Administration (FDA) and Eli Lilly and Company. JP has participated in Advisory panels or consulted with Merck, Boehringer-Ingelheim, Lundbeck, Otsuka, and Janssen. JN has participated in advisory panels or consulted with Merck, Boehringer-Ingelheim, Lundbeck, Otsuka, and Janssen; has received grant support from the NIH and the Substance Abuse and Mental Health Services Administration (SAMHSA); has served as a consultant for Alkermes, Inc., Intra-cellular Therapies, Inc., Sunovion and Merck; and served on a Data Safety Monitoring Board for Amgen. GN has received grant support from the NIH, the Barnes Jewish Hospital Foundation, the McDonnell Center for Systems Neuroscience, and Usona Institute (drug only), and has served as a consultant for Alkermes, Inc., Otsuka and Sunovion. The remaining authors declare that the research was conducted in the absence of any commercial or financial relationships that could be construed as a potential conflict of interest.

## Publisher's Note

All claims expressed in this article are solely those of the authors and do not necessarily represent those of their affiliated organizations, or those of the publisher, the editors and the reviewers. Any product that may be evaluated in this article, or claim that may be made by its manufacturer, is not guaranteed or endorsed by the publisher.
